# Investigation of the Vascular-Endothelial Pattern of Expression of DAPK-1 in Oral Squamous Cell Carcinoma and Oral Potentially Malignant Disorders Through Immunohistochemistry

**DOI:** 10.7759/cureus.63519

**Published:** 2024-06-30

**Authors:** Petros Papadopoulos, Vasileios Zisis, Dimitrios Andreadis, Konstantinos Vahtsevanos, Athanasios Poulopoulos

**Affiliations:** 1 Oral Medicine/Pathology, Aristotle University of Thessaloniki, Thessaloniki, GRC; 2 Oral and Maxillofacial Surgery, Aristotle University of Thessaloniki, Thessaloniki, GRC

**Keywords:** oral lichen planus, oral cancers, oral leukoplakia, dapk, oral potentially malignant disorders (opmd), oscc

## Abstract

Introduction

Potentially malignant disorders, like oral lichen planus (OLP) and oral leukoplakia (OL) of several degrees of dysplasia, manifest a significant potential of malignant transformation being a precursor of oral squamous cell carcinoma (OSCC). The role of microvascularization in carcinogenesis is critical; therefore, microvascularization constitutes a major therapeutic target. DAPK-1 constitutes a possible cancer marker, with proven implications in other human cancers, and there isn't any study on its vascular endothelial expression in the oral cavity, particularly in oral cancer and oral potentially malignant diseases. The present study aims to investigate the vascular endothelial expression of the DAPK-1 in paraffin-embedded tissue samples of oral leukoplakia, oral squamous cell carcinoma, and oral lichen planus.

Materials and methods

The study focuses on the immunohistochemical, vascular-endothelial, expression pattern of biomarker DAPK-1 (NBP2-38468, Novus Biologicals, Centennial, CO, US). Tissue samples were obtained from six cases of oral lichen planus (OLP) (3 of reticular and 3 of erosive form), 30 cases of oral leukoplakia (OL) (10 with no dysplasia, 10 with mild dysplasia, and 10 with moderate/severe dysplasia), 22 cases of OSCC (2 well-differentiated, 17 moderately differentiated, and 3 poorly differentiated), as well as 5 cases of normal oral epithelium. The tissue samples were retrieved from the archives of the Department of Oral Medicine/Pathology, School of Dentistry, Aristotle University of Thessaloniki, as well as from St Lukas Hospital of Thessaloniki, Greece, from 2004-2019. In accordance with the Research and Ethics Committee guidelines of the Aristotle University, School of Dentistry, and the Helsinki II declaration, the study was conducted. The primary inclusion criteria for the study focused on the presence of sufficient precancerous or cancerous tissue. Conversely, inadequate tissue served as the exclusion criteria. The staining was evaluated exclusively in a quantitative manner. The vascular endothelial staining was evaluated as either positive or negative. If at least one endothelial cell exhibited positive staining, the section was classified as positive. Statistical analysis was carried out using SPSS Statistics v25.0 (IBM Corp., Armonk, NY, US) utilizing Pearson's chi-square or Fisher's exact test, depending on the sample size, to compare OLP to OL, OLP to OSCC, OLP to normal, OL to OSCC, OL to normal, and OSCC to normal. The significance level was established at 0.05 (p=0.05).

Results

A prevalence of positive OL cases may be noticed. The comparison between OLP and OL yielded Fisher’s exact test of p>0.999, OLP and OSCC p=0.389, OLP and normal oral epithelium p>0.999, OL and OSCC p=0.226, OL and normal oral epithelium p>0.999, as well as OSCC and normal oral epithelium p=0.342.

Conclusions

The role of DAPK in tumorigenesis is already supported by limited literature. However, its implication in the development of OSCC and oral potentially malignant disorders (OPMDs) has yet to be elucidated. Its elevated expression in OL suggests a role in affecting the microenvironment, the vessels, in particular, surrounding oral potentially malignant lesions, possibly assisting their transition into cancer. The evaluation of the vascular-endothelial immunohistochemical profile of DAPK-1 in OL, OLP, and OSCC requires further studies in more tissue samples to illustrate its possible implications.

## Introduction

DAPK-1 was initially identified as a facilitator of cell death [1]. Prior research demonstrated that the downregulation of DAPK-1 expression effectively inhibited γ interferon-induced apoptotic cell death in HeLa cells (immortalized cell line). In addition, when the expression of DAPK was increased, HeLa cells perished spontaneously without any external stimuli [2,3]. The association between cancer and DAPK-1 was established through the observation of considerable methylation in the DAPK-1 promoter regions in several types of human tumors, as compared to their corresponding normal tissue samples [4-6].

Most commonly encountered tumors have a low level of DAPK-1 expression [7,8]. Factors such as p53 have the ability to directly and indirectly alter the catalytic activity and pro-apoptotic function of DAPK-1 [9,10]. DAPK-1 may trigger autophagy [6], but the specific connections between DAPK-1 and proteins associated with autophagy have not been clarified. Therefore, there may be unidentified proteins that connect DAPK-1 to the subsequent signaling pathways. The DAPK-1 gene has been observed to undergo alternative splicing, resulting in the production of two forms: a normal DAPK-1 and a variation known as s-DAPK-1 [11,12]. Alternative splicing is a crucial process in RNA maturation that enables the retention of some exons, or portions of exons and introns, in mature transcripts. This process enhances the functionality and variety of the proteome. DAPK-1 has both pro-apoptotic and anti-apoptotic properties [1,13] and a connection has been established between the expression of DAPK-1 and Fas-mediated apoptosis (Fas is a membrane protein belonging to the death receptor family. Cross-linking of Fas by its ligand, FasL, or agonistic anti-Fas antibodies, induces apoptosis of cells expressing Fas on the membrane by triggering a cascade of caspases) in human endometrial adenocarcinoma cells, shedding light on the role of DAPK-1 in promoting the survival of tumor cells [1].

The expression of DAPK-1 was reduced in HHUA cells (human cell line derived from endometrial cancer), which led to a large increase in Fas-mediated apoptosis. This suggests that endogenous DAPK acts as a negative regulator of Fas-mediated apoptosis. As a result, DAPK-1 may also play a role in promoting cell survival, depending on the specific conditions within the cell and its surrounding environment. Since DAPK-1 promotes autophagy and apoptosis, it may, in turn, inhibit tumor development and metastasis [14,15].

Tumors necessitate a vascular system for their expansion and spread through the bloodstream. Angiogenesis is a mechanism through which tumors develop their own network of small blood vessels [16]. From a structural and functional perspective, these newly formed blood vessels are not typical. They exhibit higher permeability, slower development, and the propensity to rapidly multiply [16]. The role of microvascularization in carcinogenesis is critical; therefore, the microvascularization constitutes a major therapeutic target. DAPK-1 constitutes a possible cancer marker, with proven implications in other human cancers, and there isn't any study on its vascular endothelial expression in the oral cavity, particularly in oral cancer and oral, potentially malignant diseases. The present study aims to investigate the vascular endothelial expression of the DAPK-1 in paraffin-embedded tissue samples of oral leukoplakia, oral squamous cell carcinoma, and oral lichen planus. 

## Materials and methods

The study focuses on the immunohistochemical and vascular-endothelial expression pattern of biomarker DAPK-1 (NBP2-38468, Novus Biologicals, Centennial, CO, US). The tissue samples were obtained from 6 cases of oral lichen planus (OLP) (3 of reticular and 3 of erosive form), 30 cases of oral leukoplakia (OL) (10 with no dysplasia, 10 with mild dysplasia, and 10 with moderate/severe dysplasia), 22 cases of OSCC (2 well-differentiated, 17 moderately differentiated, and 3 poorly differentiated) as well as 5 cases of normal oral epithelium. The tissue samples were retrieved from the archives of the Department of Oral Medicine/Pathology, School of Dentistry, Aristotle University of Thessaloniki, as well as from St Lukas Hospital of Thessaloniki, Greece, during the period 2004-2019. The study was conducted in accordance with the Research and Ethics Committee guidelines of Aristotle University, School of Dentistry, and the Helsinki II declaration. Approval for the present study was granted by the Ethics Committee of the School of Dentistry, Aristotle University of Thessaloniki, Greece, during its meeting on 21.11.2018, under protocol number 29/21.11.2018. The primary inclusion criteria for the study focused on the presence of sufficient precancerous or cancerous tissue. Conversely, inadequate tissue served as the exclusion criteria. The study falls within the realm of semiquantitative research. Table 1 provides a summary of the epidemiological and topographical data for the cases examined.

**Table 1:** Summary of the epidemiological and topographical data of the examined cases OL: oral leukoplakia, OSCC: oral squamous cell carcinoma, M: male, F: female

Category	Subcategory	Location	Sex	Age
Lichen planus	Reticular lichen planus	Buccal mucosa	M	61
Lichen planus	Reticular lichen planus	Buccal mucosa	M	46
Lichen planus	Reticular lichen planus	Tongue	M	70
Lichen planus	Erosive lichen planus	Tongue	M	63
Lichen planus	Erosive lichen planus	Gingiva	M	63
Lichen planus	Erosive lichen planus	Gingiva	F	50
Leukoplakia	Non-dysplastic OL	Tongue	M	69
Leukoplakia	Non-dysplastic OL	Tongue	M	53
Leukoplakia	Non-dysplastic OL	Buccal mucosa	F	58
Leukoplakia	Non-dysplastic OL	Buccal mucosa	F	55
Leukoplakia	Non-dysplastic OL	Buccal mucosa	F	61
Leukoplakia	Non-dysplastic OL	Palate	M	39
Leukoplakia	Non-dysplastic OL	Buccal mucosa	M	51
Leukoplakia	Non-dysplastic OL	Gingiva	F	60
Leukoplakia	Non-dysplastic OL	Buccal mucosa	F	58
Leukoplakia	Non-dysplastic OL	Buccal mucosa	M	55
Leukoplakia	Mildly dysplastic OL	Buccal mucosa	M	55
Leukoplakia	Mildly dysplastic OL	Gingiva	F	60
Leukoplakia	Mildly dysplastic OL	Buccal mucosa	F	58
Leukoplakia	Mildly dysplastic OL	Tongue	F	41
Leukoplakia	Mildly dysplastic OL	Buccal mucosa	M	51
Leukoplakia	Mildly dysplastic OL	Buccal mucosa	F	58
Leukoplakia	Mildly dysplastic OL	Tongue	M	69
Leukoplakia	Mildly dysplastic OL	Buccal mucosa	F	61
Leukoplakia	Mildly dysplastic OL	Buccal mucosa	M	56
Leukoplakia	Mildly dysplastic OL	Buccal mucosa	M	69
Leukoplakia	Moderately & severely dysplastic OL	Tongue	F	65
Leukoplakia	Moderately & severely dysplastic OL	Tongue	M	57
Leukoplakia	Moderately & severely dysplastic OL	Gingiva	F	62
Leukoplakia	Moderately & severely dysplastic OL	Tongue	F	70
Leukoplakia	Moderately & severely dysplastic OL	Gingiva	F	70
Leukoplakia	Moderately & severely dysplastic OL	Floor of the mouth	M	69
Leukoplakia	Moderately & severely dysplastic OL	Tongue	M	52
Leukoplakia	Moderately & severely dysplastic OL	Tongue	M	64
Leukoplakia	Moderately & severely dysplastic OL	Tongue	F	70
Leukoplakia	Moderately & severely dysplastic OL	Tongue	M	52
Oral squamous cell carcinoma	Well-differentiated OSCC	Alveolar process	F	72
Oral squamous cell carcinoma	Well-differentiated OSCC	Gingiva	F	79
Oral squamous cell carcinoma	Moderately differentiated OSCC	Buccal mucosa	M	62
Oral squamous cell carcinoma	Moderately differentiated OSCC	Alveolar process	F	56
Oral squamous cell carcinoma	Moderately differentiated OSCC	Alveolar process	F	59
Oral squamous cell carcinoma	Moderately differentiated OSCC	Tongue	F	67
Oral squamous cell carcinoma	Moderately differentiated OSCC	Alveolar process	F	75
Oral squamous cell carcinoma	Moderately differentiated OSCC	Tongue	F	65
Oral squamous cell carcinoma	Moderately differentiated OSCC	Tongue	F	27
Oral squamous cell carcinoma	Moderately differentiated OSCC	Tongue	F	79
Oral squamous cell carcinoma	Moderately differentiated OSCC	Lip	F	77
Oral squamous cell carcinoma	Moderately differentiated OSCC	Floor of the mouth	M	46
Oral squamous cell carcinoma	Moderately differentiated OSCC	Alveolar process	M	72
Oral squamous cell carcinoma	Moderately differentiated OSCC	Tongue	F	42
Oral squamous cell carcinoma	Moderately differentiated OSCC	Buccal mucosa	M	33
Oral squamous cell carcinoma	Moderately differentiated OSCC	Alveolar process	M	30
Oral squamous cell carcinoma	Moderately differentiated OSCC	Tongue	F	82
Oral squamous cell carcinoma	Moderately differentiated OSCC	Gingiva	F	78
Oral squamous cell carcinoma	Moderately differentiated OSCC	Gingiva	M	57
Oral squamous cell carcinoma	Poorly differentiated OSCC	Palate	M	35
Oral squamous cell carcinoma	Poorly differentiated OSCC	Alveolar process	M	80
Oral squamous cell carcinoma	Poorly differentiated OSCC	Alveolar process	M	80
Normal	Normal	Lip	F	79
Normal	Normal	Lip	M	44
Normal	Normal	Buccal mucosa	M	23
Normal	Normal	Buccal mucosa	F	49
Normal	Normal	Tongue	M	36

The stepwise study protocol of the immunohistochemical method included the following: fixation of the tissue sections in formalin solution 10%, embedment in paraffin blocks cut into 4-6 μm thick tissue sections using a microtome Jung Biocut 2035 (Rankin Biomedical Corporation, Davisburg, MI, US), application to electrostatically charged micro slides, deparaffinization in xylenes using three changes for 5 minutes each, gradual hydration through graded alcohols: washing in 100% ethanol twice for 15 minutes each, then 90% ethanol twice for 15 minutes each, washing in deionized H2O for 1 minute with stirring, aspiration of the excess liquid from slides, antigen retrieval by heat treatment, blocking of the endogenous peroxidase activity of the tissue, dipping of the slides in phosphate-buffered-saline (PBS) solution, application of primary antibody for 15 minutes, dipping of the slides in PBS solution, application of envision for 30 minutes, dipping of the slides in PBS solution, application of the dab envision chromogen for 15 minutes’ maximum, dipping of the slides in PBS solution, wash in deionized water for 1 minute with stirring, application of hematoxylin for 10 seconds, mounting of the sections, coverring the sections with coverslips, and finally, examination under microscope and camera.

Vascular endothelial staining was evaluated as either positive or negative. If at least one endothelial cell exhibited positive staining, the section was classified as positive. Statistical analysis was carried out using SPSS Statistics version 25.0 (IBM Corp., Armonk, NY, US) utilizing the Pearson chi-square test or Fisher's exact test, depending on the sample size. The significance level was established at 0.05 (p=0.05).

## Results

In general, the positive cases included one OLP, five OL, one OSCC, and one normal (Table 2).

**Table 2:** Vascular staining (VS), positive or negative, per tissue sample OL: oral leukoplakia, OSCC: oral squamous cell carcinoma, M: male, F: female, VS: vascular staining, +: positive, -: negative

Category	Subcategory	Location	Sex	Aage	DAPK VS
Lichen planus	Reticular lichen planus	Buccal mucosa	M	61	-
Lichen planus	Reticular lichen planus	Buccal mucosa	M	46	-
Lichen planus	Reticular lichen planus	Tongue	M	70	-
Lichen planus	Erosive lichen planus	Tongue	M	63	+
Lichen planus	Erosive lichen planus	Gingiva	M	63	-
Lichen planus	Erosive lichen planus	Gingiva	F	50	-
Leukoplakia	Non-dysplastic OL	Tongue	M	69	+
Leukoplakia	Non-dysplastic OL	Tongue	M	53	+
Leukoplakia	Non-dysplastic OL	Buccal mucosa	F	58	-
Leukoplakia	Non-dysplastic OL	Buccal mucosa	F	55	-
Leukoplakia	Non-dysplastic OL	Buccal mucosa	F	61	+
Leukoplakia	Non-dysplastic OL	Palate	M	39	-
Leukoplakia	Non-dysplastic OL	Buccal mucosa	M	51	-
Leukoplakia	Non-dysplastic OL	Gingiva	F	60	-
Leukoplakia	Non-dysplastic OL	Buccal mucosa	F	58	-
Leukoplakia	Non-dysplastic OL	Buccal mucosa	M	55	-
Leukoplakia	Mildly dysplastic OL	Buccal mucosa	M	55	-
Leukoplakia	Mildly dysplastic OL	Gingiva	F	60	-
Leukoplakia	Mildly dysplastic OL	Buccal mucosa	F	58	-
Leukoplakia	Mildly dysplastic OL	Tongue	F	41	+
Leukoplakia	Mildly dysplastic OL	Buccal mucosa	M	51	-
Leukoplakia	Mildly dysplastic OL	Buccal mucosa	F	58	-
Leukoplakia	Mildly dysplastic OL	Tongue	M	69	-
Leukoplakia	Mildly dysplastic OL	Buccal mucosa	F	61	-
Leukoplakia	Mildly dysplastic OL	Buccal mucosa	M	56	-
Leukoplakia	Mildly dysplastic OL	Buccal mucosa	M	69	-
Leukoplakia	Moderately & severely dysplastic OL	Tongue	F	65	-
Leukoplakia	Moderately & severely dysplastic OL	Tongue	M	57	-
Leukoplakia	Moderately & severely dysplastic OL	Gingiva	F	62	-
Leukoplakia	Moderately & severely dysplastic OL	Tongue	F	70	+
Leukoplakia	Moderately & severely dysplastic OL	Gingiva	F	70	-
Leukoplakia	Moderately & severely dysplastic OL	Floor of the mouth	M	69	-
Leukoplakia	Moderately & severely dysplastic OL	Tongue	M	52	-
Leukoplakia	Moderately & severely dysplastic OL	Tongue	M	64	-
Leukoplakia	Moderately & severely dysplastic OL	Tongue	F	70	-
Leukoplakia	Moderately & severely dysplastic OL	Tongue	M	52	-
Oral squamous cell carcinoma	Well-differentiated OSCC	Alveolar process	F	72	-
Oral squamous cell carcinoma	Well-differentiated OSCC	Gingiva	F	79	-
Oral squamous cell carcinoma	Moderately differentiated OSCC	Buccal mucosa	M	62	-
Oral squamous cell carcinoma	Moderately differentiated OSCC	Alveolar process	F	56	-
Oral squamous cell carcinoma	Moderately differentiated OSCC	Alveolar process	F	59	-
Oral squamous cell carcinoma	Moderately differentiated OSCC	Tongue	F	67	-
Oral squamous cell carcinoma	Moderately differentiated OSCC	Alveolar process	F	75	-
Oral squamous cell carcinoma	Moderately differentiated OSCC	Tongue	F	65	-
Oral squamous cell carcinoma	Moderately differentiated OSCC	Tongue	F	27	-
Oral squamous cell carcinoma	Moderately differentiated OSCC	Tongue	F	79	-
Oral squamous cell carcinoma	Moderately differentiated OSCC	Lip	F	77	-
Oral squamous cell carcinoma	Moderately differentiated OSCC	Floor of the mouth	M	46	-
Oral squamous cell carcinoma	Moderately differentiated OSCC	Alveolar process	M	72	-
Oral squamous cell carcinoma	Moderately differentiated OSCC	Tongue	F	42	-
Oral squamous cell carcinoma	Moderately differentiated OSCC	Buccal mucosa	M	33	-
Oral squamous cell carcinoma	Moderately differentiated OSCC	Alveolar process	M	30	-
Oral squamous cell carcinoma	Moderately differentiated OSCC	Tongue	F	82	-
Oral squamous cell carcinoma	Moderately differentiated OSCC	Gingiva	F	78	-
Oral squamous cell carcinoma	Moderately differentiated OSCC	Gingiva	M	57	-
Oral squamous cell carcinoma	Poorly differentiated OSCC	Palate	M	35	-
Oral squamous cell carcinoma	Poorly differentiated OSCC	Alveolar process	M	80	+
Oral squamous cell carcinoma	Poorly differentiated OSCC	Alveolar process	M	80	-
Normal	Normal	Lip	F	79	-
Normal	Normal	Lip	M	44	-
Normal	Normal	Buccal mucosa	M	23	+
Normal	Normal	Buccal mucosa	F	49	-
Normal	Normal	Tongue	M	36	-

The statistical analysis revealed the following: The comparison between OLP and OL yielded a Fisher’s exact test of p>0.999, OLP and OSCC p=0.389, OLP and normal oral epithelium p>0.999, OL and OSCC p=0.226, OL and normal oral epithelium p>0.999, as well as OSCC and normal oral epithelium p=0.342 (Table 3).

**Table 3:** Statistically analyzed data Intra-comparisons among six cases of oral lichen planus (OLP), 30 cases of oral leukoplakia (OL), 22 cases of oral squamous cell carcinoma (OSCC), as well as 5 cases of normal oral epithelium. The statistical significance level was set at 0.05 (p≤ 0.05).

Statistical comparisons	Associated Fisher’s exact test p values
OLP- OL	p>0.999
OLP- OSCC	p=0.389
OLP- normal	p>0.999
OL- OSCC	p=0.226
OL- normal	p>0.999
OSCC- normal	p= 0.342

**Figure 1:**
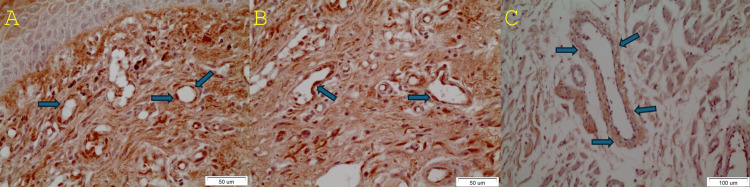
The blue arrows show the positively stained endothelial cells in two cases of oral leukoplakia (A and B) and a more diffuse pattern surrounding the endothelial cells of a vessel adjacent to an oral squamous cell carcinoma (C)

The prevalence of positive OL cases may be noticed. Furthermore, the pattern of expression in certain tissue samples may be deemed as noteworthy (Figure 1).

**Figure 2:**
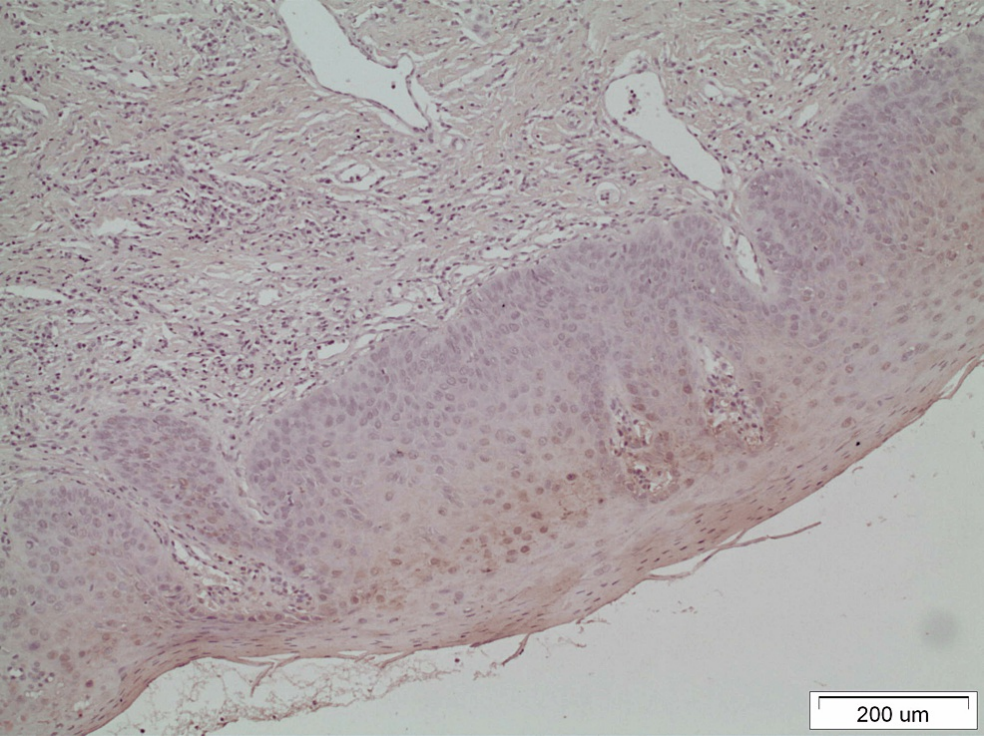
The negatively stained endothelial cells in a case of oral leukoplakia may be noticed

Figure 2 shows the negatively stained endothelial cells in a case of oral leukoplakia to illustrate the antithesis to the positively stained vessels of Figure 1.

## Discussion

Tumor-suppressor genes inhibit the growth and division of aberrant cells to maintain the appropriate cellular equilibrium. DAPK-1 is considered a tumor suppressor due to its hypermethylation and loss of expression, which have been observed in various types of malignancies, including cervical cancer [17,18]. The promoters of tumor-suppressor genes are frequently methylated, resulting in their downregulation and the advancement of cancer [17,19,20]. Multiple tumor-suppressor genes that are repressed in this manner play a role in regulating crucial biological processes such as DNA repair, cell cycle, and apoptosis [17-19]. The absence of DAPK-1 expression in cancer cell lines has been proven to result from epigenetic suppression through DNA hypermethylation [21]. DAPK-1 hypermethylation and loss of its expression have been associated with various types of cancer, including bladder, kidney, gastric, head and neck, thyroid, lung, ovarian, and cervical. In our study, there was only one positive OSCC case and the lack of vascular endothelial expression of DAPK-1 in OSCC (which constitutes a subcategory of head and neck cancer) may be attributed to this epigenetic suppression through DNA hypermethylation and the subsequent loss of expression of DAPK-1. This observation entails certain clinical implications. For example, negatively stained dysplastic lesions may already manifest this epigenetic suppression and DNA hypermethylation, thus negative expression may serve as an unfavorable prognostic factor. On the other hand, positive expression may indicate that this specific epigenetic suppression and DNA hypermethylation have not taken place, and therefore act as a favorable prognostic factor in cancerous lesions.

DAPK-1 suppression has an impact on apoptosis, which, in turn, contributes to the genesis and progression of cancer. Cells lacking DAPK-1 expression as a result of promoter hypermethylation exhibit increased invasiveness and metastatic potential [1]. There is not always a direct relationship between the methylation status and expression of the DAPK-1 gene. The use of a DNA methylation inhibitor called 5′-azadeoxycytidine (5-aza-dC) did not result in the restoration of DAPK-1 expression in certain B cell and lung cancer cell lines [21]. In addition, the absence of promoter hypermethylation may also result in a decrease in DAPK-1 expression [22]. The need for novel anticancer drugs arises due to chemotherapeutic resistance, side effects, and non-specific toxicity. Pin1 plays a critical role in the development of cancer by inhibiting tumor suppressors and promoting the activation of oncogenes [23]. DAPK-1 plays a role in the phosphorylation of Pin1, which consequently hinders its capacity to activate transcription factors associated with oncogenes. DAPK-1 may have the potential to enhance cancer treatment by inhibiting the oncogenic activity of Pin1. This could lead to the development of more effective therapeutic techniques for cancer [24,25]. Furthermore, the anticancer potential of DAPK-1 and its synergistic combination with PDCD6 in ovarian cancer was verified in a study conducted by Lee et al. [26]. In our study, the prevalence of positive OL cases was illustrated as well as a more diffuse pattern of expression surrounding the vessels underlying OSCC. The statistically nonsignificant results may be interpreted in two different ways: either the sample size did not suffice, and therefore a statistically significant correlation could not be established, or the vascular endothelial expression of DAPK remains relatively the same in normal, OL, OLP, and OSCC cases and therefore cannot serve as a prognostic factor in oral lesions.

Limitations

The limitations of the study include the lack of a TNM (tumor, nodes, and metastases) classification and five-year survival rate of the OSCC cases, as well as the lack of information regarding the course of the disease in OSCC cases and the malignant transformation rate of the OL cases involved. Furthermore, a sample size calculation did not take place due to the lack of material especially regarding the OLP cases. The authors simply used all of the available material.

## Conclusions

The role of DAPK in tumorigenesis is already supported by limited literature. However, its implication in the development of oral squamous cell carcinoma and oral potentially malignant disorders has yet to be elucidated. Our study yielded certain preliminary data, regarding its vascular-endothelial expression. Its elevated expression in OL suggests a role in affecting the microenvironment, the vessels, in particular, surrounding oral potentially malignant lesions, possibly assisting their transition into cancer. The evaluation of the vascular-endothelial immunohistochemical profile of DAPK-1 in OL, OLP, and OSCC requires further studies in more tissue samples to illustrate its possible implications.
